# Predicting Heart Failure in Patients with Atrial Fibrillation: A Report from the Prospective COOL-AF Registry

**DOI:** 10.3390/jcm12041265

**Published:** 2023-02-06

**Authors:** Rungroj Krittayaphong, Ply Chichareon, Chulalak Komoltri, Poom Sairat, Gregory Y. H. Lip

**Affiliations:** 1 Department of Medicine, Division of Cardiology, Faculty of Medicine Siriraj Hospital, Mahidol University, Bangkok 10700, Thailand; 2 Cardiology Unit, Department of Internal Medicine, Faculty of Medicine, Prince of Songkla University, Songkla 90110, Thailand; 3 Department of Research Promotion, Faculty of Medicine Siriraj Hospital, Mahidol University, Bangkok 10700, Thailand; 4 Liverpool Centre for Cardiovascular Science, University of Liverpool, Liverpool John Moores University and Liverpool Heart & Chest Hospital, Liverpool L14 3PE, UK; 5 Department of Clinical Medicine, Aalborg University, 9220 Aalborg, Denmark

**Keywords:** atrial fibrillation, heart failure, predictive risk model

## Abstract

Background: This study aimed to determine risk factors and incidence rate and develop a predictive risk model for heart failure for Asian patients with atrial fibrillation (AF). Methods: This is a prospective multicenter registry of patients with non-valvular AF in Thailand conducted between 2014 and 2017. The primary outcome was the occurrence of an HF event. A predictive model was developed using a multivariable Cox-proportional model. The predictive model was assessed using C-index, D-statistics, Calibration plot, Brier test, and survival analysis. Results: There were a total of 3402 patients (average age 67.4 years, 58.2% male) with mean follow-up duration of 25.7 ± 10.6 months. Heart failure occurred in 218 patients during follow-up, representing an incidence rate of 3.03 (2.64–3.46) per 100 person-years. There were ten HF clinical factors in the model. The predictive model developed from these factors had a C-index and D-statistic of 0.756 (95% CI: 0.737–0.775) and 1.503 (95% CI: 1.372–1.634), respectively. The calibration plots showed a good agreement between the predicted and observed model with the calibration slope of 0.838. The internal validation was confirmed using the bootstrap method. The Brier score indicated that the model had a good prediction for HF. Conclusions: We provide a validated clinical HF predictive model for patients with AF, with good prediction and discrimination values.

## 1. Introduction

Heart failure (HF) is one of the most common comorbid conditions in patients with atrial fibrillation (AF) [1,2]. Long-standing AF can lead to HF and, conversely, HF can also increase risk of AF [3]. In the Global Anticoagulant Registry in the Field-Atrial Fibrillation (GARFIELD-AF) registry, the incidence rate of HF in patients with newly diagnosed AF was 2.41 per 100 person-years, which is even greater than the incidence rate of ischemic stroke and major bleeding [4]. In Asian patients with AF, the incidence of HF was 3.01–4.25 per 100 person-years [5,6], which is perhaps higher than that reported from non-Asian data [4,7].

AF and HF have a bidirectional pathophysiological link including the development of fibrosis, structural and electrical remodeling, and neurohormonal activation [8]. They have mutual risk factors such as aging, obesity, diabetes, hypertension, and coronary artery disease (CAD). AF can lead to cardiomyopathy and HF especially during rapid ventricular response. Likewise, the increased left ventricular filling pressure can lead to high left atrial pressure and initiate or worsen the AF condition [8]. The rate control of patients with AF with and without HF is associated with a better long-term outcome [9]. The early rhythm control of patients with AF and HF has been shown to have beneficial effects on left ventricular function [10].

Recent guidelines for the management of AF have highlighted the importance of the appropriate evaluation and characterization [11] and the holistic management of AF [12], including the treatment of comorbidities, such as hypertension, diabetes, and HF [13,14].

Since the presence of AF commonly leads to incident HF, our aim was to develop a prediction model of incident HF that is especially relevant to an Asian population. A few studies have reported a predictive model for incident HF in patients with AF [15,16] and the only data from Asian population are from a Japanese population [17].

In this analysis from a contemporary nationwide prospective AF registry from Thailand, we aimed to determine the risk factors, incidence rate, and develop a predictive risk model for HF requiring hospital admission or an urgent, unscheduled clinic/office/emergency department visit in Asian patients with AF.

## 2. Methods

The Cohort of Antithrombotic Use and Optimal INR Level in Patients with Non-valvular Atrial Fibrillation in Thailand (COOL-AF Thailand) registry was a prospective nationwide multicenter study of patients with non-valvular AF aged more than 18 years. The exclusion criteria were as follows: (i) rheumatic mitral valve disease; (ii) prosthetic mechanical valve; (iii) inability to have a follow-up visit; (iv) life expectancy less than 3 years; (v) AF from transient reversible cause; (vi) ischemic stroke of less than 3 months; (vii) hematologic disease that increased the risk of bleeding such as myeloproliferative disease; and (viii) refusal to participate.

The study was approved by the Central Research Ethics Committee (CREC) with the Certificate of Approval number COA-CREC 003/2014. Written informed consent was obtained prior to participation.

### 2.1. Study Protocol

After informed consent, the baseline data were collected from the medical records and patient interviews. All the data were written in the case record form and transferred into the web-based system. The case record forms were sent to the data management unit where all the data were verified. Queries were created when any questions arose during the verification process. The follow-up data were collected every 6 months in a similar manner. The site monitoring was performed for every study site to ensure the data quality.

### 2.2. Data Collection

The following baseline data were collected: demographic, weight, height, details of AF, medical history, concomitant diseases, physical examination, medications, laboratory data, ECG and investigational lab data, and components of CHA_2_DS_2_-VASc and HAS-BLED score. For follow-up visits, the data were recorded similar to the baseline visit but also included the clinical outcome data.

### 2.3. Study Outcomes

The main outcome in this study was the occurrence of an HF event. A HF event was defined as a hospital admission or a presentation of the patient for an urgent, unscheduled clinic/office/emergency department visit, with a primary diagnosis of HF, whereby the patient exhibits new or worsening symptoms of HF on presentation, has objective evidence of new or worsening HF, and receives initiation or intensification of treatment specifically for HF [18]. The investigators were required to upload essential documentation to support the diagnosis of HF into the web-based system. All the supporting documents in the web system were sent to the adjudication committee to confirm the diagnosis.

### 2.4. Statistical Analysis

The continuous data were presented as mean and standard deviation (SD) and compared between 2 groups with the student *t*-test for unpaired data. The categorical data were presented as number and percentages and compared using the chi-square test. The univariable and multivariable analysis was performed to identify the factors that predict the HF outcome using the Cox proportional Hazard model. A univariable analysis was performed using all the baseline variables. The variables with *p*-values < 0.05 in the univariable analysis were selected for multivariable analysis using backward elimination with *p*-values < 0.05 as the stopping criteria. Since the data of this study were prospectively collected, the data were relatively completed. The only variable that had missing data is the left ventricular ejection fraction (LVEF) from an echocardiogram, which was missing in 512 cases (15%). We applied imputation for LVEF for the missing data to solve the issue.

### 2.5. Model Development

The risk prediction model for HF was developed from the variables derived from the multivariable analysis. The probability of HF at 3 years for each patient was calculated by using the following equation that is derived from the Cox proportional hazards model:

P_Heart failure_ at 3 years = 1 − S_0_(t)^exp (Prognostic Index)^

where S_0_(t) is the average survival probability at time t (i.e., at 3 years), prognostic index is the sum of the predictor product, and the coefficient obtained from the multivariable analysis.

### 2.6. Model Validation

The assessments of the risk of bias and concerns regarding model applicability were performed according to the Prediction model Risk of Bias Assessment Tool (PROBAST) suggestion [19]. Bootstrapping was used to verify the fitted model, based on 100 bootstrap samples. The calibration was tested using the calibration slope based on the observed and predicted hazards of HF. A calibration slope of close to 1 shows a good agreement [20]. The C-statistics were used to measure the model discrimination. The C-statistics vary from 0 to 1 and the value close to 1 indicates a good prediction model [21]. A Receiving Operating Characteristics (ROC) curve was used to compare the C-statistics. The D-statistics assess the discrimination ability of the fitted model into low-risk and high-risk patients [21]. The D-statistic would be 0 or higher; if the D-statistic was 0, it can be concluded that this risk prediction model is not capable of discriminating. The C- and D- statistics were also calculated after the bootstraps as an internal validation. The apparent and optimism-corrected values of the C- and D-statistics were reported. The Cox proportional Hazard model was also used to test the differences in the cumulative event rate between the patients who belong to different risk groups according to the predictive model. The Brier score was evaluated to assess the predictive ability of the model [22]. The Net Reclassification Index (NRI) and Integrated Discrimination Index (IDI) was performed based on the previously proposed methods to determine the influence of the predictive model on the reclassification of the study subjects [23]. Decision-curve analyses (DCA) were used to estimate the clinical usefulness of the HF prediction models by assessing the ability to make better decisions with a model than without [24].

A sensitivity analysis was performed for the assessment of the model for the prediction of HF in those who did not have a history of HF to test the benefit of the model to predict new HF cases. The sensitivity analysis was also performed on the complete dataset after the removal of patients with missing LVEF data. Additional analysis was performed to test the COOL-AF prediction model that was created from the COOL-AF population after the removal of patients with a history of heart failure or LVEF < 50%. The COOL-AF model was compared with other models.

The predictive model of the COOL-AF study was compared with 3 previously reported models from the Framingham Heart Study [16], ORBIT-AF study [15], and a study from Japan [17]. Since 2 previous studies [15,17] reported variables in the multivariable analysis and did not report the regression model, we fitted all the variables in the multivariable analysis into the regression model for comparison purposes. One study [16] did report the regression model but it was the model for the 10-year risk of HF. Therefore, we fitted the reported variables for 3-year HF risk for comparison.

All the statistics were performed using the SPSS statistical software version 18.0 (SPSS, Inc., Chicago, IL, USA) and R version 3.6.3 (www.r-project.org, accessed on 1 March 2020).

## 3. Results

**Figure 1:**
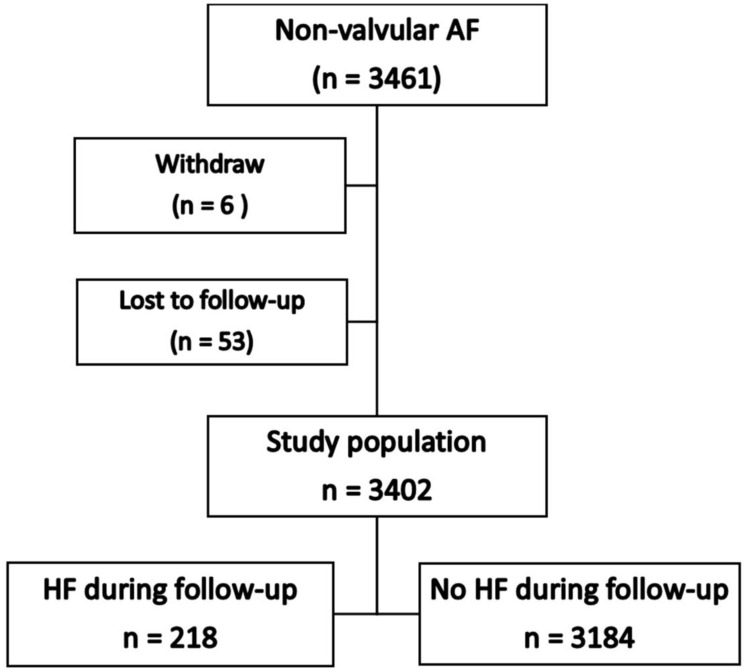
Flow diagram of study population.

We included a total of 3402 patients in this analysis (Figure 1): mean age 67.4 ± 11.3 years, 1980 (58.2%) male. The average follow-up duration was 25.7 ± 10.6 months or 7912 person-years, during which HF developed during follow-up in 218 patients (6.4%). The incidence rate of HF was 3.03 (2.64–3.46) per 100 person-years. The baseline characteristics of the study population with and without HF outcome are shown in Table 1. There were significant differences between the patients with and without HF in age, proportion of females, and various comorbidities.

### 3.1. Type of Heart Failure at Baseline and During Follow-Up

A history of HF was present in 912 patients (26.8%). Echocardiography data were available in 834 patients (91.4%). Among the patients with HF who had echocardiographic data, the HF phenotypes at baseline were as follows: heart failure with reduced ejection fraction (HFrEF) 241 (28.9%), heart failure with mildly reduced ejection fraction (HFmrEF) 127 (15.2 %), and heart failure with preserved ejection fraction (HFpEF) 466 (55.9%).

Among the 218 patients who developed HF during follow-up, 204 patients (93.6%) had echocardiographic data during the HF event. The patients (%) with HFrEF, HFmrEF, and HFpEF were 56 (27.0%), 21 (9.8%), and 127 (63.2%), respectively.

### 3.2. Univariable and Multivariable Analysis

The univariable and multivariable analyses were performed to determine the factors predicting HF (Table 2). On multivariable analysis, independent predictors of HF requiring hospital admission or an urgent, unscheduled clinic/office/emergency department visit were as follows: old age, female, history of HF, history of coronary artery disease (CAD), cardiac implantable electronic devices (CIED), hypertension, diabetes, smoking, renal replacement therapy, and LVEF < 50%.

### 3.3. Model Development

Based on the significant association of factors predicting the HF outcome from a multivariable model, we can predict the 3-year HF risk for each COOL-AF patient using the following equation:

P_Heart failure at 3 years_ = 1 − 0.98805554^exp (Prognostic Index)^

where the Prognostic Index = 0.533626 × Age ≥ 65 years + 0.554760 × Female gender + 0.850436 × History of heart failure + 0.514349 × History of coronary artery disease + 0.433954 × Cardiac implantable electronic device + 0.711797 × Diabetes mellitus + 0.379917 × Hypertension + 0.541620 × Smoking + 1.427287 × Renal replacement therapy + 0.569514 × LVEF < 50%.

### 3.4. Model Validation

The C-index was 0.756 (95% CI: 0.723–0.789). The D-statistic was 1.532 (95% CI: 1.303–1.761), which indicated that the model can be used to discriminate the patients with a high risk or low risk of incident HF. When compared to the low-risk group according to the prediction model, the high-risk group had a Hazard Ratio of 6.90 (4.37–10.92).

The patients were categorized into five groups (very low, low, moderate, high, and very high risk) according to their quintile of predicted probability of HF derived from the score.

**Figure 2:**
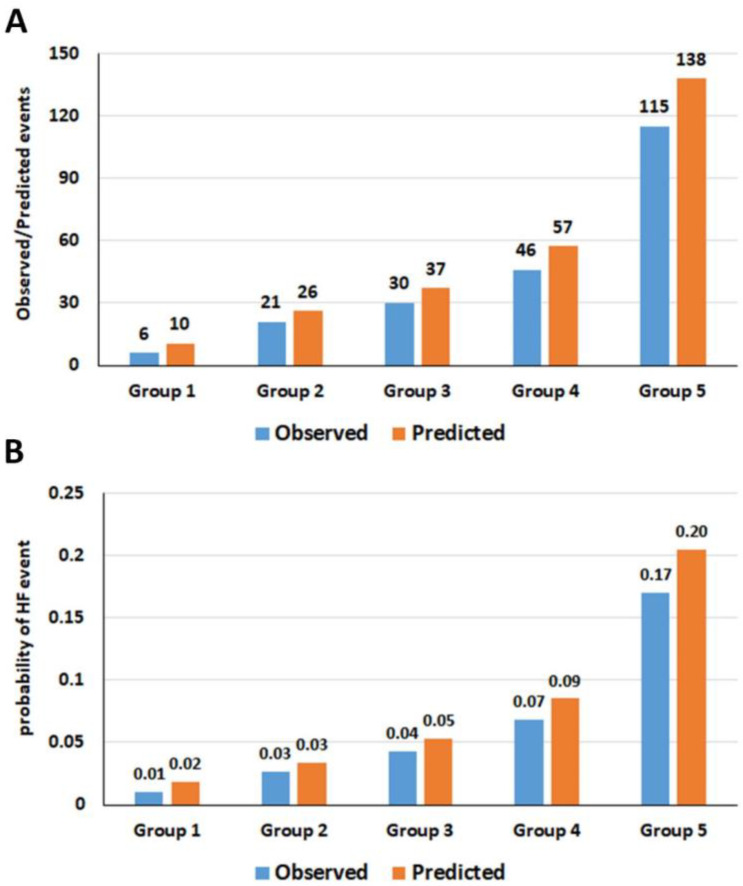
Bar graph showing agreement of observed and predicted HF events (**A**) and probability of HF events (**B**).

**Figure 3:**
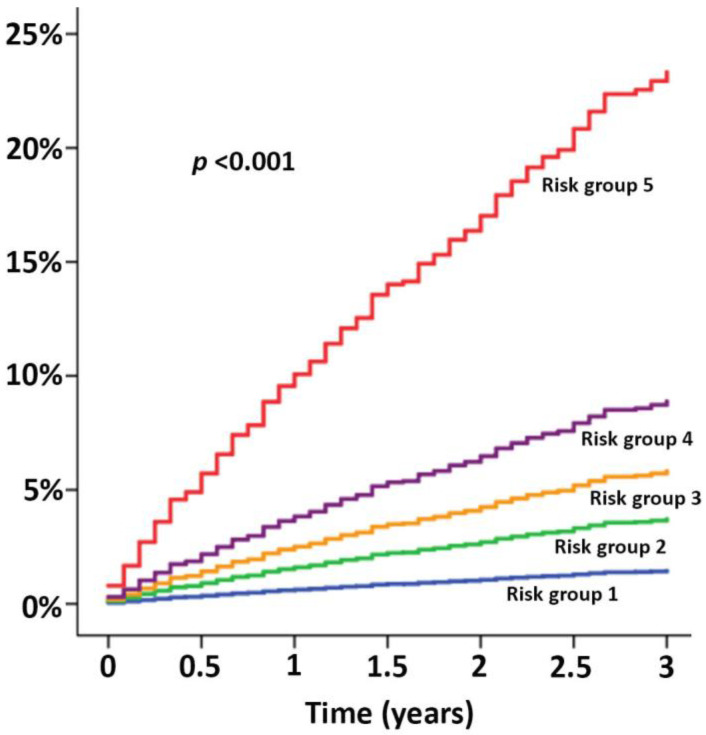
Hazard graph showing the risk of HF events during the 3-year follow-up stratified by the risk groups from very low risk (Risk group 1) to very high-risk (Risk group 5).

**Figure 4:**
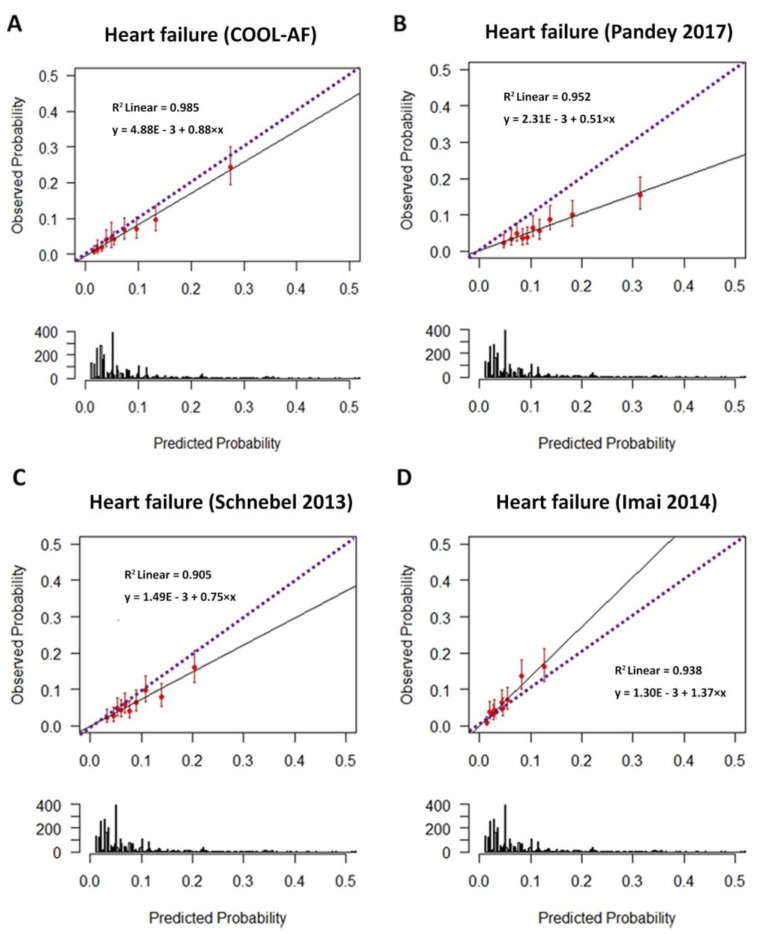
Calibration plots showing a good agreement of the probability of predicated and observed HF events among 10 equal groups of four predictive models. (**A**) COOL-AF; (**B**) Pandey et al. [15]; (**C**) Schnabel et al. [16] and (**D**) Imai et al. [17].

The agreement between the observed and predicted HF event and percentage of probability for HF is shown in Figure 2. The cumulative HF event rates over time in the patients belong to different risk groups are shown in Figure 3, which demonstrates well-separated Hazard graphs between each risk group. The calibration plots showed good agreement between predicted probability derived from the regression model and actual outcomes among 10 groups of patients with a calibration slope of 0.838 and the intercept of −0.002 (Figure 4A).

For internal validation, we performed 100 bootstraps. The C- and D-statistics after the bootstraps were 0.756 (0.737–0.775) and 1.503 (1.372–1.634). The Brier score was 0.056, which indicated that the model can predict the HF event very well.

The optimisms for the C-statistics, slope, and intercept of the calibration plot were calculated using the bootstrap method. The optimisms were 0.019, 0.045, and 0.105 for the C-statistics, slope, and intercept, respectively. After correcting the optimism, the C-statistics, slope, and intercept of the calibration plot were 0.747 (0.745–0.750), 0.836, and −0.100, respectively.

The sensitivity analysis for the assessment of model prediction in patients who did not have a history of HF showed that the C-statistics remain high (0.700 95% CI 0.682–0.718). The R^2^ of the calibration plot was 0.933 and the NRIs compared to the other three models were 10.07–29.75%. The sensitivity analysis was also performed on the complete dataset after the removal of the patients with missing LVEF data. The results showed that all the variables in the multivariable analysis model remained in the multivariable model of the complete dataset with the exception of female gender. There was additional analysis of the results of the COOL-AF prediction model with the exclusion of 1147 patients with a history of heart failure or LVEF < 50%. The results demonstrated that the C-statistics remain high (0.712, 95% CI 0.693–0.731), which is superior to the other three models that had C-statistics ranging from 0.630 to 0.690. The R^2^ of the calibration plot was 0.803 and the COOL-AF model NRIs compared to the other three models were 4.49–26.90%.

### 3.5. Simplified HF Prediction Score

We developed a simplified HF prediction score using a coefficient of 10 variables in the prediction model. For a simplified HF score, Age > 65 years = 1; Female gender = 1; History of heart failure = 2; History of coronary artery disease = 1; Cardiac implantable electronic device = 1; Diabetes mellitus = 2; Hypertension = 1; Smoking = 1; Renal replacement therapy = 3; and LVEF < 50% = 1. The total score = 14. The patients in this cohort had scores varying from 0 to 11. The C-statistics of the simplified HF prediction score in the prediction of HF at 3 years was 0.751 (95% CI = 0.736–0.766), which is equivalent to the C-statistics of the original model that was 0.757 (0.742–0.771). The incidence rate and HF event rate at 3 years increased as the score increased. The calibration plot of the model using a simplified HF prediction score and observed probability and HF prediction model showed a good agreement (Supplementary Figure S1). Supplementary Table S1 and Supplementary Figure S2A show the incidence rate per 100 person-years and rate of HF at 3 years using a simplified HF prediction score.

We classified the HF risk according to the simplified HF score as low risk (score 0–2), intermediate risk (score 3–5), high risk (score 6–7), and very high risk (score ≥ 8). The incidence rate for HF and the 3 years HF event rate increased from low-risk to very high risk with the 3 years event rate at 2% in the low-risk group and 34.8% in very-high-risk group (Supplementary Figure S2B,C).

### 3.6. Comparisons of COOL-AF Predictive Model with Previous Studies

**Figure 5:**
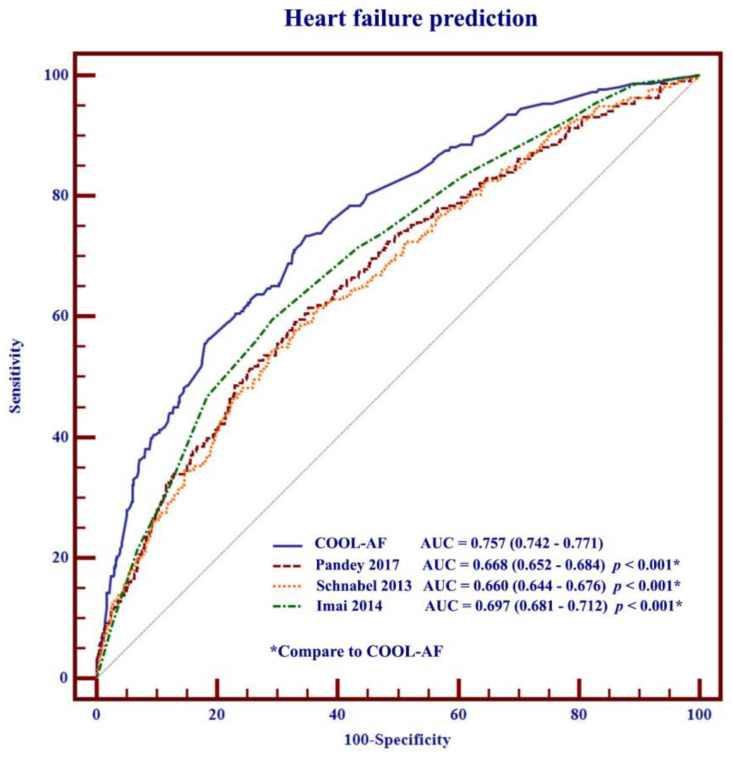
Comparisons of C-statistics of COOL-AF model with Pandey et al. [15], Schnabel et al. [16], and Imai et al. [17] (AUC = area under the curve). * Statistical significance.

**Figure 6:**
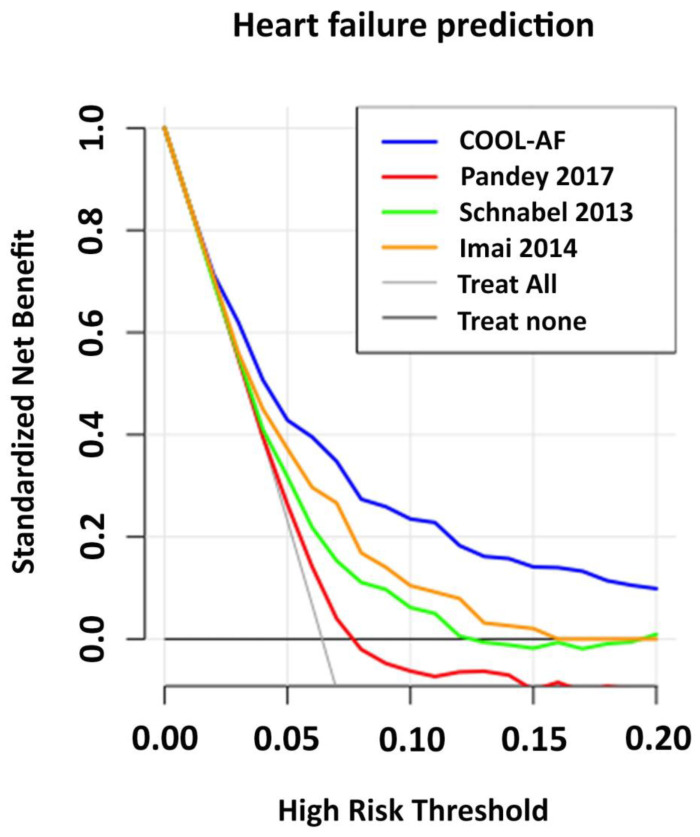
Decision curve analysis (DCA) for the assessment of the ability to make better decisions with a model than without. Each graph represents DCA of each model compared to no models (treat all and treat none). COOL-AF model was compared to Pandey et al. [15], Schnabel et al. [16] and Imai et al. [17].

The predictive model of the COOL-AF study was compared to the models derived from the variables of the predictive models from the three previous studies as mentioned in the statistical section (Section 2.4). All the predictive models are shown in Supplementary Table S2. Supplementary Table S3 shows coefficients of variables in the models of COOL-AF and the other three studies. The calibration plot of the COOL-AF model was closer to the identity line when compared to the calibration plot of the previous studies (Figure 4). The C-statistics of the COOL-AF model were significantly higher than the other models (*p* < 0.001) (Figure 5). The results of the NRI and IDI analysis demonstrated a better reclassification of cases and controls using the COOL-AF model compared to the other three models (Supplementary Figure S3) with the NRI ranging from 31.8% to 59.5%. The decision curve analysis showed a better net number of true positives gained using the COOL-AF model compared to the other three models and no model at different threshold probabilities (Figure 6). The 3-year HF risk prediction can be applied with mobile application (Supplementary Figure S4).

### 3.7. Additional Analysis to Identify Preventive Strategies to Reduce HF Risk

We performed analysis to determine whether better symptom management, cardiovascular risk factors, and comorbidities management, which are the B and C components of the AF better care (ABC) pathway [12], and a good control of blood pressure and heart rate can reduce HF risk. The results showed that the incidence rate of patients with a B compliant, as defined by the European Heart Rhythm Association (EHRA), score ≤ 2 had an incidence rate of HF 2.43 (2.03–2.89) compared to those with B non-compliant [5.59 (4.48–6.88)], *p* < 0.001. Similarly, the patients with C-compliant, as defined by the appropriate management of cardiovascular risk factors and comorbidities, had a lower incidence rate of HF compared to those with C non-compliant [2.56 (2.12–3.06) vs. 4.39 (3.56–5.35), *p* < 0.001]. The patients who had systolic blood pressure (SBP) within the target defined by the SBP 120–140 mmHg had a lower rate of HF compared with those with SBP out of target [2.68 (2.12–3.34) vs. 3.50 (2.94–4.13), *p* = 0.029]. The patients with resting heart rates < 100 beats/minute had a lower rate of HF compared with those with HRs ≥ 100 beats/minute [2.68 (2.12–3.34) vs. 3.50 (2.94–4.13), *p* = 0.029].

## 4. Discussion

The results of this prospective multicenter registry of patients with non-valvular AF in Thailand demonstrated that the incidence of HF was 3.03 (2.64–3.46) per 100 person-years. Second, the predictive factors of HF were age >65 years, female gender, history of HF, history of CAD, CIED, diabetes mellitus, hypertension, smoking, renal replacement therapy, and LVEF < 50%. Third, the model can be used to discriminate the patients with a high risk or low risk of incident HF, with good calibration after internal validation. After the data input, the HF risk can be easily calculated using a mobile application (Supplementary Figure S2) and for those who are at high or very high risk, preventive strategies can be initiated to reduce the risk of HF hospitalization.

The incidence rate of HF in patients with AF in our study was 3.03 per 100 person-years, which was higher than the results of the ORBIT-AF study, which is 1.8 per 100 person-years where the majority of patients were non-Asians [15]. In other studies, a HF incidence rate of 1.83 per 100 person-years has been reported from the ACTIVE, RE-LY, and AVERREOS trial populations [25]. The data on the HF phenotypes of our study also showed that, among patients with incident HFrEF, HfmrEF, and HfpEF, the highest rates were evident in HfpEF, which is in agreement with the prior studies showing the close AF and HFpEF [26].

The Asian population has been shown to have more HF burden than non-Asian populations [27]. Another explanation for the different HF incidence rates in the literature is that AF management may have been changed and many guidelines now focus also on the risk factor and comorbidity management in AF patients [13,28]. Previous studies demonstrated disparities in AF management especially with regard to the use of oral anticoagulant (OAC) and the quality of OAC control [29,30].

The COOL-AF HF prediction model, compared to the results of three previous studies, demonstrated that the C-statistics of the COOL-AF model were higher than other models and that the NRI was in the range from 31.8% to 59.5%. The calibration plots showed good agreement between the predicted probability derived from the regression model and actual outcomes among 10 groups of patients with a calibration slope of 0.838. The HF prediction model of Pandey et al. and Schnabel et al. tends to overestimate the HF risk while the model from Imai et al. tends to underestimate the HF risk. Besides, the results of Schnabel et al. and Imai et al. were based on a small sample size (725 and 347 patients) [16,17]. In our AF cohort, the important predictors of incident HF were >65 years, female gender, history of HF, history of CAD, CIED, diabetes mellitus, hypertension, smoking, renal replacement therapy, and low LVEF. These predictors in our study are broadly similar to previous reports [15,17,25,31,32]. Our study indicates that female gender is a predictor for HF, which differs from previous studies [33]. The data from a Japanese AF population demonstrated that females had a higher risk of HF than males and the incidence rate of incident HF in both sexes remained high [5]. In our study, the results of the sensitivity analysis after the removal of patients with a history of HF and those with LVEF < 50% showed that the C-statistics of the COOL-AF HF prediction model remained high and greater than the C-statistics from the other three models from previous studies [15,16,17].

The results of our study have some potential clinical applications. The simplified HF prediction score that we proposed on Supplementary Figure S2 can help identify those with a low risk of a 3-year HF event (2%) and a very high risk with an HF event rate of 34.8%. For the HF prediction model, if we applied the model as a mobile application, after the data input, we could calculate the absolute HF risk of each individual patient. For those who are at a high HF risk, physicians can apply some preventive strategies such as maintaining good blood pressure and heart rate control or cardiovascular risk factors and co-morbidity management that might reduce the HF risk.

We also identified some preventive strategies to reduce HF risk from the analysis of our data. These preventive strategies included better symptom management, better management of cardiovascular risk factors and comorbidities, which are the B and C components of the ABC pathway [12], and control of SBP within the target of 120–140 mmHg and heart rate < 100 beats/minute.

### Limitations

This study has some limitations. First, the majority of the patients were enrolled from large hospitals. Therefore, the results may not be generalizable to the whole group of AF patients. Second, despite the reminder to all the investigators to enroll consecutive cases, there might be some possibility of selection bias. Third, the definition of heart failure outcome may be different among different studies. In this study, we tried to use the most recommended definition for HF outcome [18]. Fourth, for the comparison of the COOL-AF model with other models, there might be some limitations since the collected variables may be different among studies. Therefore, the confounding factors that depend on the baseline data may have some differences and might impact the results of the comparison despite the attempt to refit the previous models in the COOL-AF population. Fifth, despite internal validation being performed, it would be better if there was external validation.

## 5. Conclusions

The incidence rate of HF requiring hospital admission or an urgent, unscheduled clinic/office/emergency department visit in patients with AF was 3.03 per 100 person-years. We also provide a validated clinical HF predictive model for patients with AF, with good prediction and discrimination values.

## Figures and Tables

**Table 1:** Baseline characteristics of patients with and without heart failure during follow-up.

Variables	All (*n* = 3402)	Heart Failure during Follow-Up (*n* = 218)	No Heart Failure during Follow-Up (*n* = 3184)	*p*-Value
Age (years)	67.4 ± 11.3	70.3 ± 10.8	67.2 ± 11.3	<0.001 *
Female gender	1422 (41.8%)	106 (48.6%)	1316 (41.3%)	0.035 *
Time after diagnosis of AF (years)	3.4 ± 4.3	3.9 ±5.1	3.3 ± 4.3	0.097
Atrial fibrillation				0.177
Paroxysmal	1148 (33.7%)	61 (28.0%)	1087 (34.1%)	
Persistent	643 (18.9%)	45 (20.6%)	598 (18.8%)	
Permanent	1611 (47.4%)	122 (51.4%)	1499 (47.1%)	
Symptomatic AF	2618 (77.0%)	165 (75.7%)	2453 (77.0%)	0.646
History of heart failure	912 (26.8%)	115 (52.8%)	797 (25.0%)	<0.001 *
History of CAD	547 (16.1%)	73 (33.5%)	474 (14.9%)	<0.001 *
CIED	341 (10.0%)	37 (17.0%)	304 (9.5%)	<0.001 *
History of ischemic stroke/TIA	592 (17.4%)	37 (17.0%)	555 (17.4%)	0.863
Hypertension	2328 (68.4%)	177 (81.2%)	2151 (67.6%)	<0.001 *
Diabetes mellitus	839 (24.7%)	97 (44.5%)	742 (23.3%)	<0.001 *
Smoking	678 (19.9%)	59 (27.1%)	619 (19.4%)	0.006 *
Dyslipidemia	1915 (56.3%)	146 (67.0%)	1769 (55.6%)	0.001 *
Renal replacement therapy	40 (1.2%)	10 (4.6%)	30 (0.9%)	<0.001 *
Dementia	29 (0.9%)	3 (1.4%)	26 (0.8%)	0.385
History of bleeding	323 (9.5%)	28 (12.8%)	295 (9.3%)	0.081
CHA_2_DS_2_-VASc score				<0.001 *
Low risk	287 (8.4%)	3 (1.4%)	284 (8.9%)	
Intermediate risk	546 (16.0%)	16 (7.3%)	530 (16.6%)	
High risk	2569 (75.5%)	199 (91.3%)	2370 (74.4%)	
HAS-BLED score				<0.001 *
0	490 (14.4%)	15 (6.9%)	475 (14.9%)	
1–2	2373 (69.8%)	130 (59.6%)	2243 (70.4%)	
≥3	539 (15.8%)	73 (33.5%)	466 (14.6%)	
CKD	2051 (60.3%)	161 (73.9%)	1890 (59.4%)	<0.001 *
Anemia	1286 (37.8%)	120 (55.0%)	1166 (36.6%)	<0.001 *
LVEF < 50%	618 (18.2%)	72 (33.0%)	546 (17.1%)	<0.001 *
Antiplatelet	890 (26.2%)	87 (39.9%)	803 (25.2%)	<0.001 *
Anticoagulant	2566 (75.4%)	168 (77.1%)	2398 (75.3%)	0.561
Warfarin	2338 (68.7%)	159 (72.9%)	2179 (68.4%)	0.166
NOACs	228 (6.7%)	9 (4.1%)	219 (6.9%)	0.116
Beta blocker	2476 (72.8%)	165 (75.7%)	2311 (72.6%)	0.319
CCB	934 (27.5%)	65 (29.8%)	869 (27.3%)	0.419
Digitalis	539 (15.8%)	39 (17.9%)	500 (15.7%)	0.392
MRA	280 (8.2%)	27 (12.4%)	253 (7.9%)	0.021 *
Statin	2012 (59.1%)	156 (71.6%)	1856 (58.3%)	<0.001 *
ACEI/ARB	1555 (45.7%)	119 (54.6%)	1436 (45.1%)	0.007 *

Data presented as mean ± standard deviation or number and percentage. A *p*-value < 0.05 indicates statistical significance. * Statistical significance

**Table 2:** Univariate and multivariable analysis of predicting factors for heart failure event.

Variable	Univariate Analysis		Multivariable Analysis	
	HR (95% CI)	*p*-Value	HR (95% CI)	*p*-Value
Age ≥ 65	1.82 (1.35–2.46)	<0.001 *	1.69 (1.24–2.31)	0.001 *
Female gender	1.34 (1.02–1.74)	0.033 *	1.77 (1.29–2.43)	<0.001 *
Atrial fibrillation				
Paroxysmal	Reference	0.064		
Persistent	1.36 (0.92–1.99)	0.122		
Permanent	1.44 (1.06–1.97)	0.021 *		
Symptomatic AF	0.95 (0.70–1.29)	0.737		
History of heart failure	2.94 (2.25–3.84)	<0.001 *	2.36 (1.76–3.17)	<0.001 *
History of CAD	2.69 (2.03–3.57)	<0.001 *	1.68 (1.25–2.27)	0.001 *
CIED	1.66 (1.17–2.37)	0.005 *	1.55 (1.08–2.23)	0.018 *
History of ischemic stroke/TIA	1.00 (0.70–1.43)	0.998		
Diabetes mellitus	2.50 (1.91–3.27)	<0.001 *	2.02 (1.53–2.66)	<0.001 *
Hypertension	1.97 (1.40–2.77)	<0.001 *	1.46 (1.02–2.08)	0.038 *
Smoking	1.39 (1.03–1.87)	0.031 *	1.74 (1.23–2.47)	0.002 *
Dyslipidemia	1.62 (1.22–2.16)	0.001 *		
Renal replacement therapy	4.89 (2.59–9.22)	<0.001 *	4.20 (2.19–8.04)	<0.001 *
Dementia	1.57 (0.50–4.91)	0.437		
History of bleeding	1.35 (0.91–2.01)	0.138		
History of bleeding	1.30 (0.87–1.95)	0.198		
CKD	1.91 (1.41–2.58)	<0.001 *		
Anemia	2.12 (1.63–2.77)	<0.001 *		
LVEF < 50%	2.36 (1.78–3.12)	<0.001 *	1.77 (1.29–2.44)	<0.001 *
Antiplatelet	1.79 (1.36–2.34)	<0.001 *		
Anticoagulant	1.15 (0.84–1.58)	0.378		
Warfarin	1.27 (0.94–1.72)	0.115		
NOACs	0.61 (0.31–1.18)	0.142		
Beta blocker	1.17 (0.86–1.60)	0.315		
CCB	1.16 (0.87–1.55)	0.321		
Digitalis	1.13 (0.80–1.60)	0.489		
MRA	1.63 (1.09–2.44)	0.017 *		
Statin	1.82 (1.36–2.45)	<0.001 *		
ACEI/ARB	1.43 (1.09–1.87)	0.009 *		

Data presented as mean ± standard deviation or number and percentage. A *p*-value < 0.05 indicates statistical significance. * Statistical significance

## Data Availability

The dataset that was used to support the results and conclusion of this study are included within the manuscript. The additional data are available from corresponding author upon reasonable request.

## Supplementary Materials

The following supporting information can be downloaded at: https://www.mdpi.com/article/10.3390/jcm12041265/s1, Figure S1: Calibration plot of A. Predicted probability from Simplified HF prediction model versus Observed probability and B. Predicted probability from Simplified HF prediction model versus Predicted probability from Complete HF prediction model; Figure S2: A. Heart failure (HF) event rate at 3 years stratified by simplified HF risk score. B and C. Incidence rate of HF and HF event rate at 3 years according to risk groups classified by the simplified risk score. (Simplified HF score, Age > 65 years = 1; Female gender = 1; History of heart failure = 2; History of coronary artery disease = 1; Cardiac implantable electronic device = 1; Diabetes mellitus = 2; Hypertension = 1; Smoking = 1; Renal replacement therapy = 3; and Left ventricular ejection fraction < 50% = 1); Figure S3: Net reclassification index (NRI) and Integrated Discrimination Index (IDI) of COOL-AF predictive model compared to three previous models. A. 2 by 2 table showing comparison of NRI of COOL-AF model compared to the others and category-free NRI and IDI with 95% confidence interval. B. Scattered plot of predictive probability of COOL-AF model on Y-axis compared to previous model; Figure S4: Simulation of mobile application for clinical use of COOL-AF model. A. 3-year heart failure risk based on 10 variables derived from COOL-AF population B. Output of calculation and the risk levels C. Formula used for heart failure risk calculation when left ventricular ejection fraction (LVEF) data are available (Formula 1) and not available (Formula 2) D. Example of a case E. 3-year heart failure risk of this case with LVEF data F. 3-year heart failure risk of this case when LVEF data is not available; Table S1: Incidence rate and risk of heart failure (HF) at 3 years of patients with atrial fibrillation stratified by simplified HF prediction score; Table S2: Predictive model of COOL-AF and predictive models that were derived from 3 previous studies that were refitted in COOL-AF population; Table S3: Coefficients of variables in the models of COOL-AF and the other 3 studies

## Abbreviations

AF, atrial fibrillation; CAD, coronary artery disease; CIED, cardiac implantable electronic device; TIA, transient ischemic attack; CKD, chronic kidney disease; LVEF, left ventricular ejection fraction; NOAC, non-vitamin K antagonist oral anticoagulants; CCB, calcium channel blocker; MRA, mineralocorticoid receptor antagonist; ACEI, angiotensin converting enzyme inhibitor; and ARB, angiotensin receptor blocker.
