# Supplementary material for: Predicting Heart Failure in Patients with Atrial Fibrillation: A Report from the Prospective COOL-AF Registry
Source: J Clin Med. 2023 Feb 6;12(4):1265. doi: 10.3390/jcm12041265 (PMC9967148; doi:10.3390/jcm12041265)

Supplementary materials

Supplementary Table S1. Incidence rate and risk of heart failure (HF) at 3 years of patients with atrial fibrillation stratified by simplified HF prediction score

Simplified HF prediction score	Number of patients	Number of events	100-person years	Rate per 100-person years	3-year HF event rate
0	133	1	2.66	0.38	1.19%
1	396	4	7.91	0.51	1.85%
2	653	19	13.03	1.46	2.85%
3	729	23	15.16	1.52	4.39%
4	572	36	12.46	2.89	6.73%
5	440	41	9.40	4.36	10.25%
6	228	29	4.80	6.04	15.45%
7	158	34	3.12	10.89	22.93%
8	71	22	1.33	16.56	33.25%
9	13	3	0.24	12.29	46.59%
10	6	3	0.08	38.71	62.22%
11	3	3	0.02	138.46	77.92%
**Total**	**3402**	**218**	**70.22**	**3.10**	**6.41%**

Supplementary Table S2. Predictive model of COOL-AF and predictive models that were derived from 3 previous studies that were refitted in COOL-AF population.

$P_{\text{Heart failure at 3 years}} = 1 - 0.98805554^{\exp\text{ (Prognostic Index)}}$<br>where Prognostic Index = 0.533626*Age ≥65 years + 0.554760* Female gender + 0.850436* History of heart failure + 0.514349*History of coronary artery disease + 0.433954*Cardiac implantable electronic device + 0.711797*Diabetes mellitus + 0.379917*Hypertension + 0.541620*Smoking + 1.427287*Renal replacement therapy + 0.569514*LVEF <50%.
$P_{\text{Heart failure at 3 years}}$ (Pandey et al) $= 1 - 0.99364819^{\exp\text{ (Prognostic Index)}}$<br>where Prognostic Index = 0.019148*Age (years) + 0.902666* coronary artery disease + 0.330726* renal dysfunction + 0.210616* Persistent AF + 0.328719* Permanent AF + 0*Paroxysmal AF + 0.011096* Heart rate (beats/min)
$P_{\text{Heart failure at 3 years}}$ (Schnabel et al) $= 1 - 0.99644817^{\exp\text{ (Prognostic Index)}}$<br>where Prognostic Index = 0.030844* Age (years) + 0.030916* Body mass index + 0.314508* Left ventricular hypertrophy + 0.817760* Diabetes + 0.458910* Prevalent myocardial infarction
$P_{\text{Heart failure at 3 years}}$ (Imai et al) $= 1 - 0.98498905^{\exp\text{ (Prognostic Index)}}$<br>where Prognostic Index = 0.518770* Age ≥72 years + 0.213591* Heart rate ≥80 beats/min + 0.588433*Hypertension + 1.075795*Previous history of congestive heart failure

AF = atrial fibrillation, LVEF = left ventricular ejection fraction

Supplementary Table S3. Coefficients of variables in the models of COOL-AF and the other 3 studies.

Variable	COOL-AF		Pandey et al		Schnabel et al		Imai et al	
	Coefficient	HR (95% CI)	Coefficient	HR (95% CI)	Coefficient	HR (95% CI)	Coefficient	HR (95% CI)
Elderly	0.534	1.71 (1.25-2.33)						
Female gender	0.555	1.74 (1.27-2.39)						
Congestive heart failure	0.85	2.34 (1.75-3.13)					1.076	2.93 (2.25-3.83)
Symptomatic CAD	0.514	1.67 (1.24-2.26)	0.928	2.53 (1.90-3.36)				
CIED	0.434	1.54 (1.07-2.22)						
Diabetes	0.712	2.04 (1.54-2.69)			0.818	2.27 (1.72-2.99)		
Hypertension	0.380	1.46 (1.02-2.09)					0.588	1.80 (1.28-.54)
Smoke	0.542	1.72 (1.22-2.43)						
Renal replacement therapy	1.427	4.17 (2.18-7.98)						
Renal dysfunction			0.331	1.39 (1.00-1.93)				
LVEF <50	0.570	1.77 (1.29-2.43)						
Age (Year)			0.019	1.02 (1.01-1.03)	0.031	1.03 (1.02-1.05)		
Age ≥72							0.519	1.68 (1.28-2.20)
Paroxysmal			Ref.	Ref.				
Persistent			0.211	1.23 (0.84-1.82)				
Permanent			0.329	1.39 (1.01-1.90)				
Heart rate			0.011	1.01 (1.00-1.02)				
Heart rate ≥80							0.214	1.24 (0.95-1.62)
BMI					0.031	1.03 (1.00-1.06)		
LVH					0.315	1.37 (0.91-2.07)		
History of MI or unstable angina					0.459	1.58 (1.02-2.45)		

HR = Hazard ratio, CI = confidence interval, CAD = coronary artery disease, CIED = cardiac implantable electronic devices, LVEF = left ventricular ejection, fraction, BMI = body mass index, LVH = left ventricular hypertrophy, MI = myocardial infarction

Supplementary Figure S1. Calibration plot of A. Predicted probability from Simplified HF prediction model versus Observed probability and B. Predicted probability from Simplified HF prediction model versus Predicted probability from Complete HF prediction model

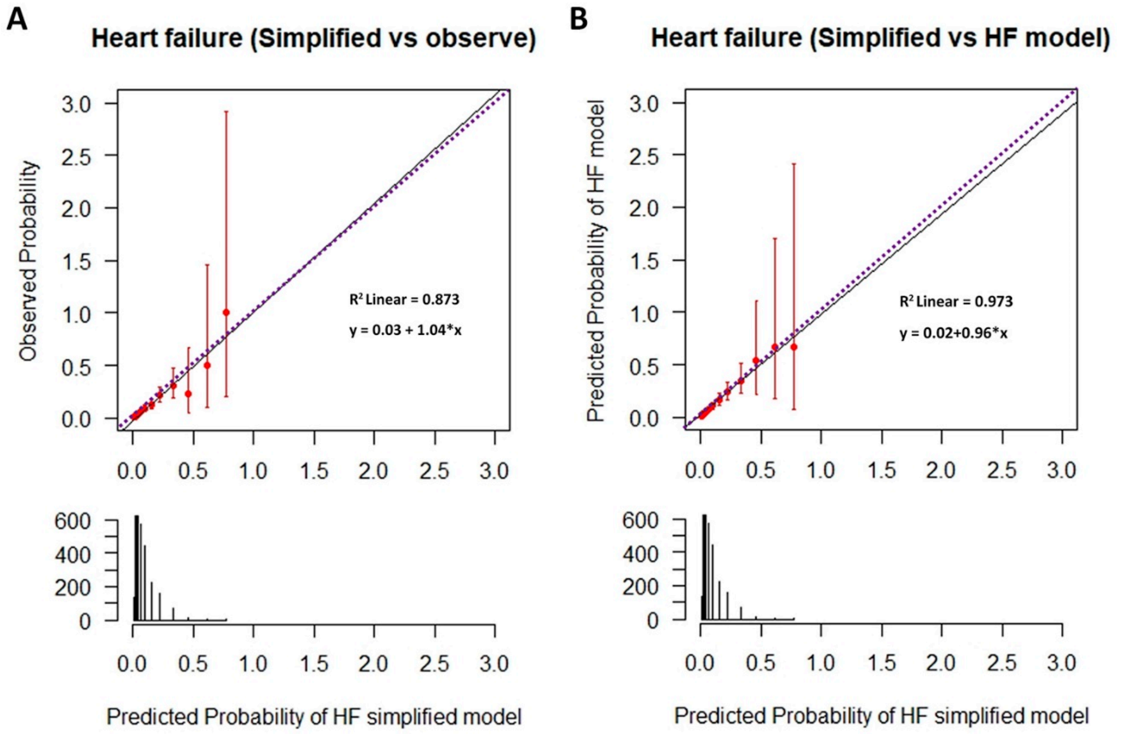

Supplementary Figure S2. A. Heart failure (HF) event rate at 3 years stratified by simplified HF risk score. B and C. Incidence rate of HF and HF event rate at 3 years according to risk groups classified by the simplified risk score. **(**Simplified HF score, Age >65 years = 1; Female gender = 1; History of heart failure = 2; History of coronary artery disease = 1; Cardiac implantable electronic device = 1; Diabetes mellitus = 2; Hypertension = 1; Smoking = 1; Renal replacement therapy = 3; and Left ventricular ejection fraction <50% = 1).

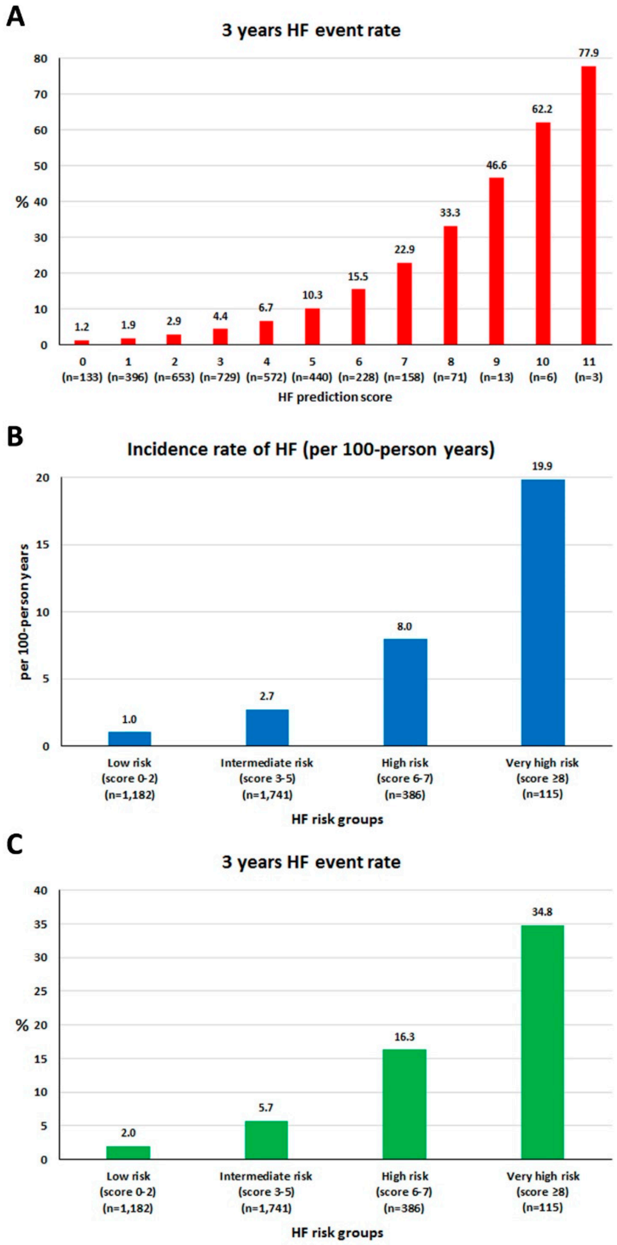

Supplementary Figure S3. Net reclassification index (NRI) and Integrated Discrimination Index (IDI) of COOL-AF predictive model compared to three previous models. A. 2 by 2 table showing comparison of NRI of COOL-AF model compared to the others and category-free NRI and IDI with 95% confidence interval. B. Scattered plot of predictive probability of COOL-AF model on Y-axis compared to previous model.

A

	$P_{M+}>P_M$	$P_{M+}<P_M$
No heart failure	561	2623
Heart failure	95	123

M+ = COOL-AF

M = Pandey et al

Green = Favor model M+ ; Pink = Favor model M

NRI (%)	IDI (%)
46.58 (33.08 - 60.76)	3.20 (1.36 – 5.04)

	$P_{M+}>P_M$	$P_{M+}<P_M$
No heart failure	847	2337
Heart failure	128	90

M+ = COOL-AF

M = Schnabel et al

Green = Favor model M+ ; Pink = Favor model M

NRI (%)	IDI (%)
59.53 (44.72 – 72.46)	5.87 (4.17 – 7.58)

	$P_{M+}>P_M$	$P_{M+}<P_M$
No heart failure	2240	944
Heart failure	185	33

M+ = COOL-AF

M = Imai et al

Green = Favor model M+ ; Pink = Favor model M

NRI (%)	IDI (%)
31.78 (21.96 – 40.80)	6.67 (4.92 – 8.43)

B

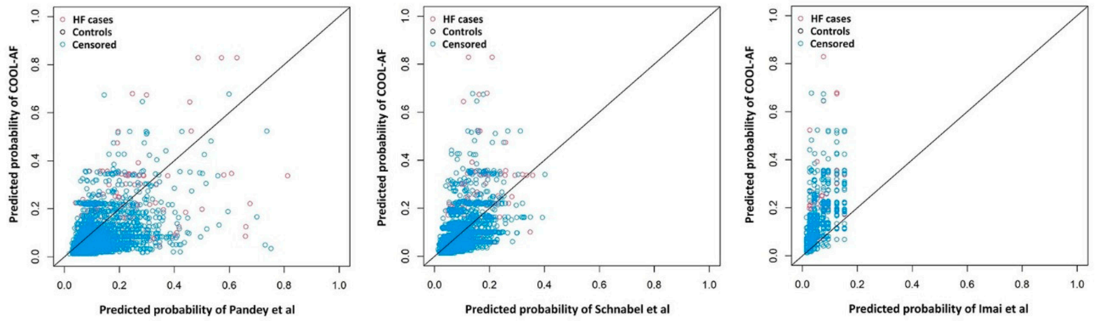

Supplementary Figure S4. Simulation of mobile application for clinical use of COOL-AF model. A. 3-year heart failure risk based on 10 variables derived from COOL-AF population B. Output of calculation and the risk levels C. Formula used for heart failure risk calculation when left ventricular ejection fraction (LVEF) data are available (Formula 1) and not available (Formula 2) D. Example of a case E. 3-year heart failure risk of this case with LVEF data F. 3-year heart failure risk of this case when LVEF data is not available

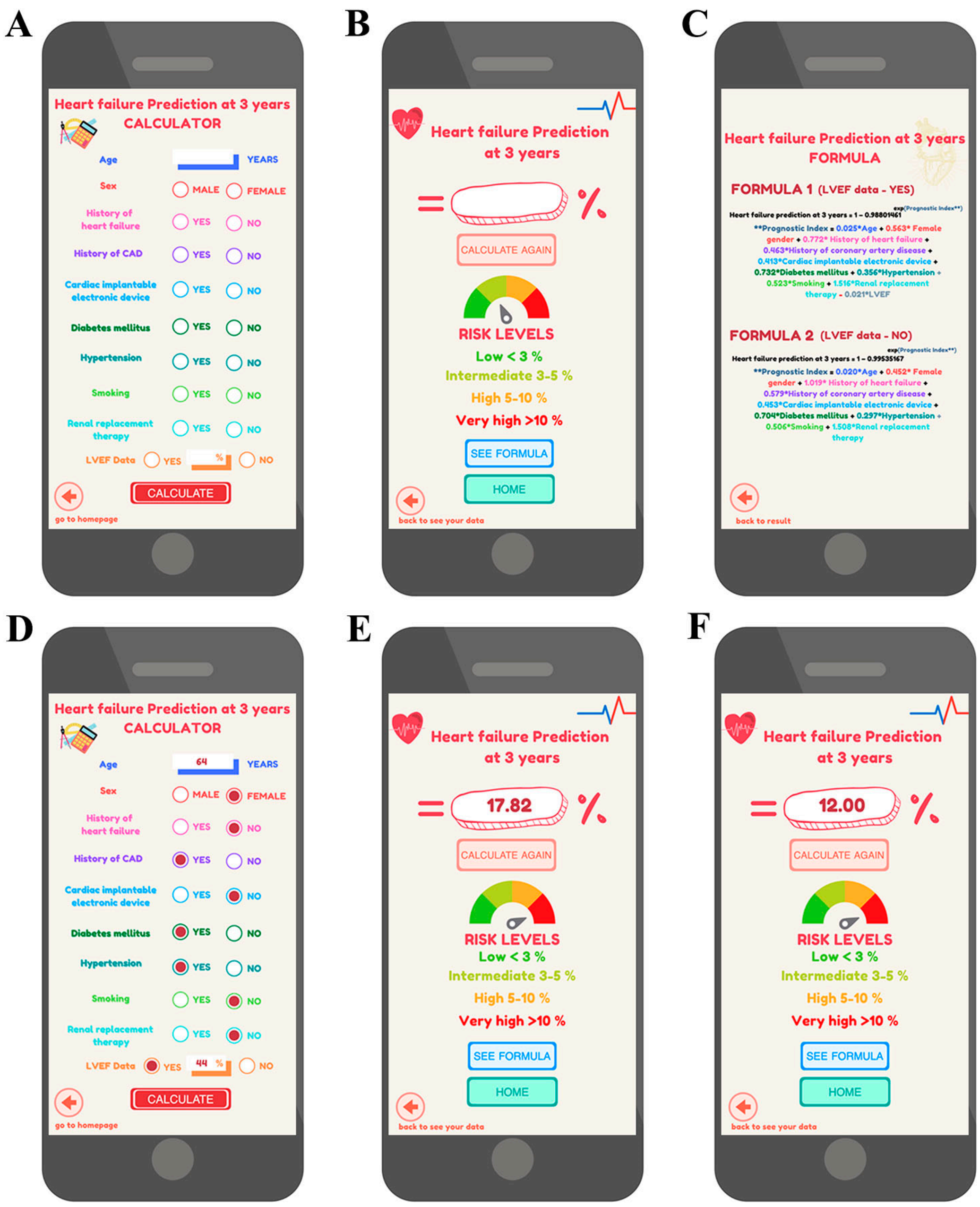

Supplement: Supplementary file 1 [file jcm-12-01265-s001.zip › jcm-2159867-supplementary.pdf]
